# Correlation between Hand Grip Strength and Peak Inspiratory Flow Rate in Patients with Stable Chronic Obstructive Pulmonary Disease

**DOI:** 10.3390/diagnostics12123050

**Published:** 2022-12-05

**Authors:** Apisara Suriyakul, Narongkorn Saiphoklang, Igor Barjaktarevic, Christopher B. Cooper

**Affiliations:** 1 Division of Pulmonary and Critical Care Medicine, Department of Internal Medicine, Faculty of Medicine, Thammasat University, Pathum Thani 12120, Thailand; 2 Division of Pulmonary, Critical Care and Sleep Medicine, Department of Medicine, David Geffen School of Medicine, University of California Los Angeles, Los Angeles, CA 90095, USA; 3 Airway & Exercise Physiology Research Laboratory, Department of Physiology, David Geffen School of Medicine, University of California Los Angeles, Los Angeles, CA 90095, USA

**Keywords:** Accuhaler, dry powder inhalers, hand grip strength, peak inspiratory flow rate, Turbuhaler

## Abstract

Optimal peak inspiratory flow rate (PIFR) is required for effective drug delivery to distal airways when using dry powder inhalers (DPIs). This study aimed to examine the association between PIFR and hand grip strength (HGS) in stable COPD patients. A cross-sectional study was conducted. PIFR was measured using the In-check DIAL to assess for Accuhaler and Turbuhaler DPIs. HGS was measured using a handheld dynamometer. A PIFR of <60 L/min was considered suboptimal PIFR. Demographics, clinical data, and spirometric data were collected and compared. Eighty-one patients (86% men) were included. Mean age was 73.3 ± 8.9 years. FEV_1_ was 65.3 ± 23.7%. The prevalence of suboptimal PIFR was 38% and 59% for Accuhaler and Turbuhaler, respectively. HGS in the suboptimal PIFR group was lower than in the optimal PIFR group for Accuhaler (22.8 ± 4.7 vs. 33.2 ± 6.9 kg, *p* < 0.001) and for Turbuhaler (25.3 ± 6.4 vs. 35.1 ± 6.3 kg, *p* < 0.001). The equation for predicted Accuhaler PIFR (L/min) was −30.340 + (0.274 × hand grip strength) − (0.206 × age) + (0.219 × height) + (1.019 × FVC). The equation for predicted Turbuhaler PIFR (L/min) was 56.196 + (0.321 × hand grip strength) − (0.196 × female) − (0.224 × age) + (0.304 × FVC). The best cutoff values of HGS for predicting optimal PIFR in Accuhaler and Turbuhaler were 26.8 kg (with 82% sensitivity and 84% specificity) and 31.9 kg (with 79% sensitivity and 90% specificity), respectively. In conclusion, HGS correlated with PIFR in patients with clinically stable COPD, especially in the group with pronounced symptoms without frequent exacerbations. HGS threshold values associated with suboptimal PIFR were identified. HGS may be used as an alternative tool to assess an optimal inspiratory force for DPIs.

## 1. Introduction

Chronic obstructive pulmonary disease (COPD) is a common cause of death worldwide, and the prevalence of the disease continues to rise [1,2]. The goals for treatment of stable COPD are to reduce current symptoms and future risks of exacerbation. Pharmacological therapy for COPD is used to reduce symptoms, reduce the frequency and severity of exacerbations, and improve exercise tolerance and health status [1]. Inhaled drugs are the cornerstone for prevention of COPD exacerbation and hospitalization [2]; they provide better pulmonary bioavailability, lower dose requirement, and less systemic toxicities than the oral or injectable drugs [3,4]. Aerosol delivery is a function of the dose deposited at the appropriate site in the lung. Aerosol deposition is affected by the amount of aerosol produced and the particle characteristics, ventilatory pattern, and airway anatomy and geometry. One of the factors associated with the ventilatory pattern that affects aerosol deposition is the inspiratory flow rate [5]. Therefore, patient inhaler techniques must be taken into account when assessing distal lung deposition.

Generation of an effective peak inspiratory flow rate (PIFR) is thought to be necessary for optimal drug delivery to distal airways and lung parenchyma, and better clinical outcomes in those on inhaler therapy, especially dry powder inhaler (DPI) users [6,7,8]. Using an arbitrary threshold of 60 L/min, several previous studies have investigated suboptimal PIFR in COPD patients with or without exacerbation, revealing an incidence of suboptimal PIFR of 30–60% [3,9,10,11], which might lead to the failure of COPD treatment. Some studies that also investigated factors associated with suboptimal PIFR showed that female sex was an associated factor [9,10,11].

Generation of inspiratory flow is dependent on thoracic geometry and inspiratory muscle force [12,13]. Thus, reduced muscular strength in general may be a problem for DPI use. Muscular strength assessed by hand grip strength (HGS) is a simple measure of upper limb muscle function and is associated with mortality in the general population and in COPD patients [14,15,16]. HGS is significantly associated with Accuhaler PIFR in hospitalized COPD patients with acute exacerbation [3].

Although PIFR directly assesses a patient’s ability to use inhalation therapy, it may not always be fully informative, especially in situations such as acute exacerbations of COPD, where differentiating acute PIFR compromise from chronic inability to properly use inhalers may not be possible. Thus, additional assessment tools that could help assess the potential of the respiratory system to create enough negative inspiratory pressures for adequate lung drug deposition are needed. We hypothesized that HGS values correlate with PIFR, allowing this test to be used as an alternative tool to assess an optimal inspiratory force for DPIs in patients with clinically stable COPD.

## 2. Materials and Methods

### 2.1. Study Design

A cross-sectional study was conducted at a medical outpatient department of Thammasat University Hospital, Thailand, between January 2021 and December 2021. COPD patients aged 40 years or older whose diagnoses were confirmed with spirometry (post-bronchodilator force expiratory volume in 1 second (FEV_1_)/forced vital capacity (FVC) < 70%) were included. Exclusion criteria were history of COPD exacerbation within 3 months, history of oral or intravenous corticosteroid treatment within 6 weeks, inability to use the devices for PIFR and HGS assessment, patients with tracheostomy, and patients requiring invasive or noninvasive mechanical ventilation. Demographics, smoking history, comorbidities, respiratory symptoms assessed by the modified Medical Research Council (mMRC) dyspnea scale [17] and COPD Assessment Test (CAT) [18], and spirometry data, including FEV_1_ and FVC, were collected.

### 2.2. Procedures

PIFR was measured by In-check DIAL^®^ (Clement Clarke International Ltd., Harlow, UK). In-Check DIAL^®^ was developed to assess PIFR [19,20]. The resistance of this device was set to simulate the Accuhaler and Turbuhaler DPIs because these DPI devices were commonly used by our COPD patients. A patient performed three consecutive measurements of PIFR for each DPI. All patients were instructed to inhale as fast as possible after a complete exhalation in a sitting position with one-minute breaks between attempts [3]. The maximal value of PIFR from three consecutive measurements of each DPI was recorded for the final analysis. The optimal PIFR for Accuhaler and Turbuhaler was ≥ 60 L/min [8]. A PIFR < 60 L/min was considered as suboptimal PIFR.

HGS was measured by Jamar^®^ hand dynamometer (Asimow Engineering Co., CA, USA) and was reported in kilograms. A patient performed the test at rest in sitting position with the dominant hand unsupported, the wrist in neutral position, the elbow at 90° flexion, and the shoulder in adduction. All patients were instructed to squeeze the hand dynamometer as hard as possible for 3–5 s. The test was performed with three attempts, with one-minute breaks between attempts [3]. The maximal value of the three efforts was recorded for the final analysis.

### 2.3. Outcomes

The primary outcome was the correlation between HGS and PIFR in clinically stable COPD patients. The secondary outcomes were prevalence of suboptimal PIFR for Accuhaler and Turbuhaler in these patients and the best cutoff value of HGS to predict optimal PIFR.

### 2.4. Statistical Analysis

In a previous study [3], hospitalized COPD patients with suboptimal PIFR had lower HGS (24.2 ± 11.1 vs. 30.9±10.9 kg) compared to those with optimal PIFR. We hypothesized that our study would find a difference in HGS between the suboptimal and the optimal PIFR groups similar to that study [3]. The sample size was calculated for a 2-sample means test using 80% power and 5% type I error. Thus, the calculated sample size would be 88. 

Descriptive statistics are presented as number (%) and mean ± standard deviation. The chi-squared test was used to compare categorical variables between the suboptimal and optimal PIFR groups. Student’s t-test was used to compare continuous variables between the two groups. Pearson correlation was used to determine the correlation between HGS and PIFR. To determine the set of variables associated with PIFR, we used the linear regression model with PIFR set as the dependent variable. All independent variables—age, sex, height, respiratory rate, FEV_1_, FVC, CAT and mMRC scores, HGS, and short-acting bronchodilator (SABD) dose—were entered into the regression model, followed by backward selection using a *p*-value cutoff of 0.1. We report the regression coefficients, their 95% confidence interval, and corresponding *p*-values. Variables with *p*-value < 0.05 were considered statistically associated with PIFR. Using the regression coefficients and the intercept, predicted PIFR for a patient could be calculated from the following equation, where V was the covariate, β was the regression coefficient, and I was the number of variables:

Predicted PIFR = intercept + V1β1 + V2β2 + Viβi

The Receiver Operator Characteristic (ROC) curve was used to determine the best HGS cutoff value to predict the optimal PIFR. A two-sides *p*-value < 0.05 was considered statistically significant. Statistical analyses were performed using SPSS version 25.0 software (IBM corp., Armonk, NY, USA).

## 3. Results

### 3.1. Participants

**Figure 1:**
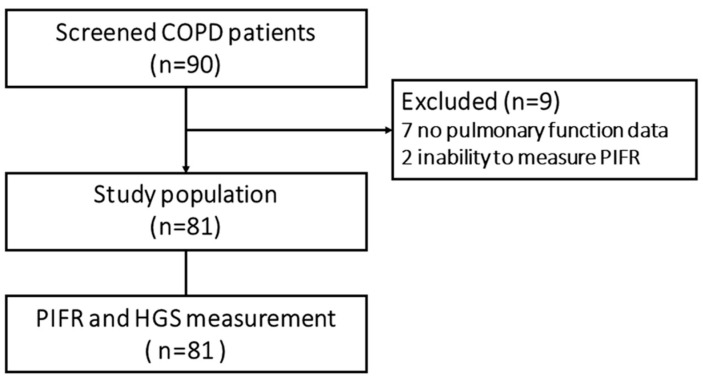
Flowchart of COPD patient recruitment to the study.

Ninety patients with clinically stable COPD were screened. Eighty-one patients were included in the study (Figure 1). Eighty-six percent were men. Mean age was 73.3 ± 8.9 years. Most patients were classified as COPD Grade A or B, as well as the Global Initiative for Chronic Obstructive Lung Disease (GOLD) spirometric stage 1. Mean post-bronchodilator FEV_1_/FVC was 58 ± 11%. Mean maximal PIFR was 63.7 ± 18.9 L/min and 52.4 ± 15.4 L/min for Accuhaler and Turbuhaler, respectively. Mean HGS was 29.2 ± 8.0 kg (Table 1).

### 3.2. Prevalence of Suboptimal PIFR

The prevalence of suboptimal PIFR, defined as PIFR <60 L/min, was 38% and 59% for Accuhaler and Turbuhaler, respectively (Table 2). Differences in PIFR and other variables between the optimal and the suboptimal PIFR groups for Accuhaler and Turbuhaler are shown in Table 2. The suboptimal groups had significantly lower PIFR than the optimal groups for Accuhaler and Turbuhaler (Table 2).

When compared to the optimal PIFR groups, the suboptimal Accuhaler and Turbuhaler groups had had more females, older patients, higher breathing frequency, more inhaled SABD use, higher CAT and mMRC scores, lower HGS, shorter height, and worse pulmonary function (Table 2).

### 3.3. Association between HGS and PIFR

HGS showed highly significant positive correlation with Accuhaler PIFR (r = 0.591, *p* < 0.001) and also with Turbuhaler PIFR (r = 0.614, *p* < 0.001) (Table 3). 

**Figure 2:**
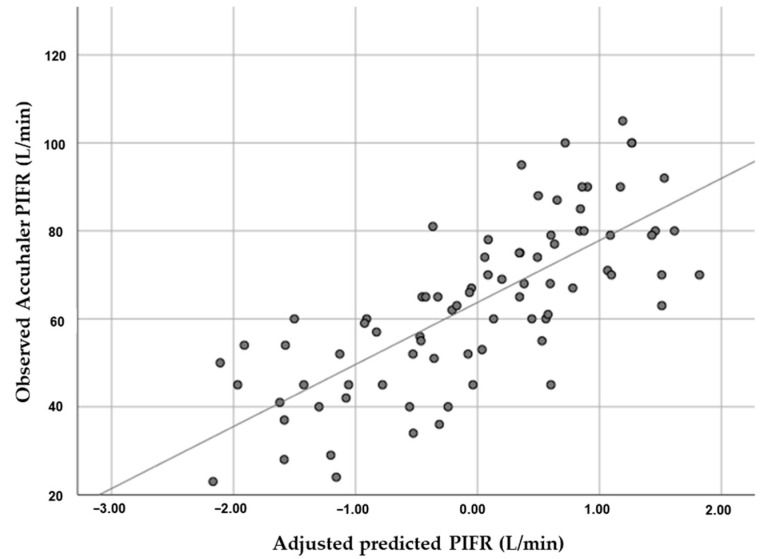
Linear regression analysis showed the correlation between peak inspiratory flow rate (PIFR) for Accuhaler and hand grip strength (HGS) adjusted by age, height, forced vital capacity (FVC), and short-acting bronchodilator dose. Predicted Accuhaler PIFR (L/min) = −30.340 + (0.274 × hand grip strength) − (0.206 × age) + (0.219 × height) + (1.019 × FVC).

The equation for predicting Accuhaler PIFR, derived from the linear regression model, predicted Accuhaler PIFR (L/min) = −30.340 + (0.274 × hand grip strength) − (0.206 × age) + (0.219 × height) + (1.019 × FVC) (Table 4 and Figure 2).

**Figure 3:**
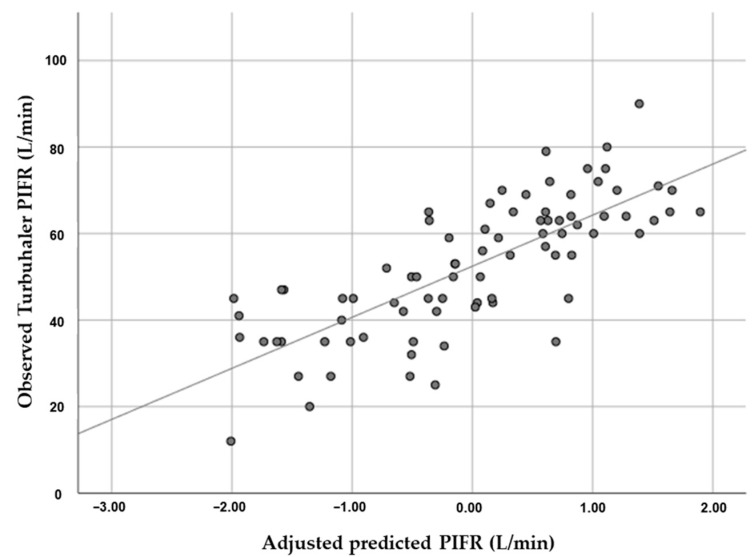
Linear regression analysis showed the correlation between peak inspiratory flow rate (PIFR) for Turbuhaler and hand grip strength (HGS) adjusted by gender, age, forced vital capacity (FVC), and short-acting bronchodilator dose. Predicted Turbuhaler PIFR (L/min) = 56.196 + (0.321 × hand grip strength) − (0.196 × female) − (0.224 × age) + (0.304 × FVC).

The equation for predicting Turbuhaler PIFR, derived from the linear regression model, predicted Turbuhaler PIFR (L/min) = 56.196 + (0.321 × hand grip strength) − (0.196 × female) − (0.224 × age) + (0.304 × FVC) (Table 4 and Figure 3).

### 3.4. HGS Cutoff Value for Predicting Optimal Accuhaler and Turbuhaler PIFR

**Figure 4:**
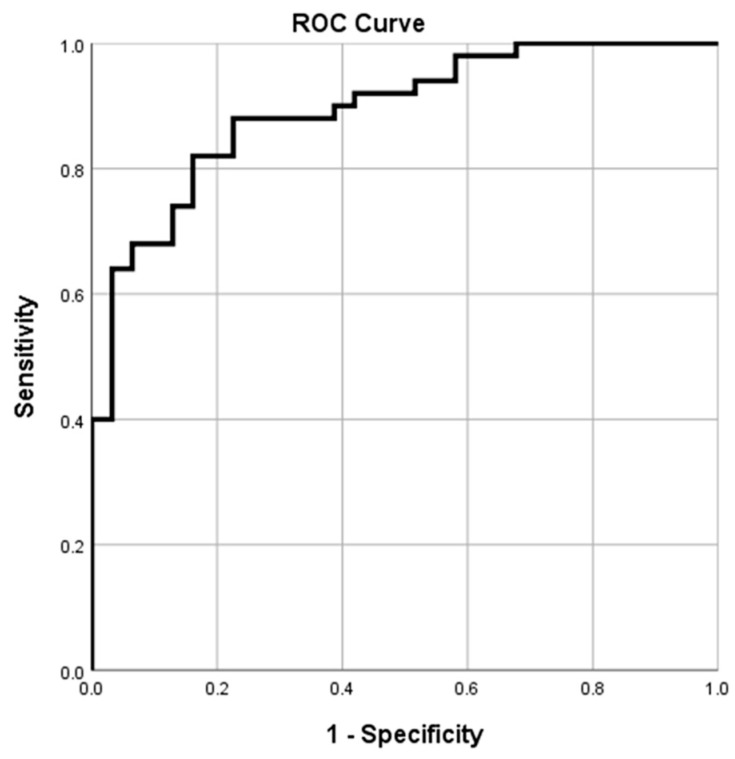
The receiver operating characteristic (ROC) plot of handgrip strength (HGS) and peak inspiratory flow rate (PIFR) for Accuhaler. The best cutoff value of HGS for Accuhaler is 26.8 kg, with the area under the ROC curve of 0.892 (95% CI; 0.824–0.961, *p* <0.001), 82% sensitivity, and 84% specificity.

**Figure 5:**
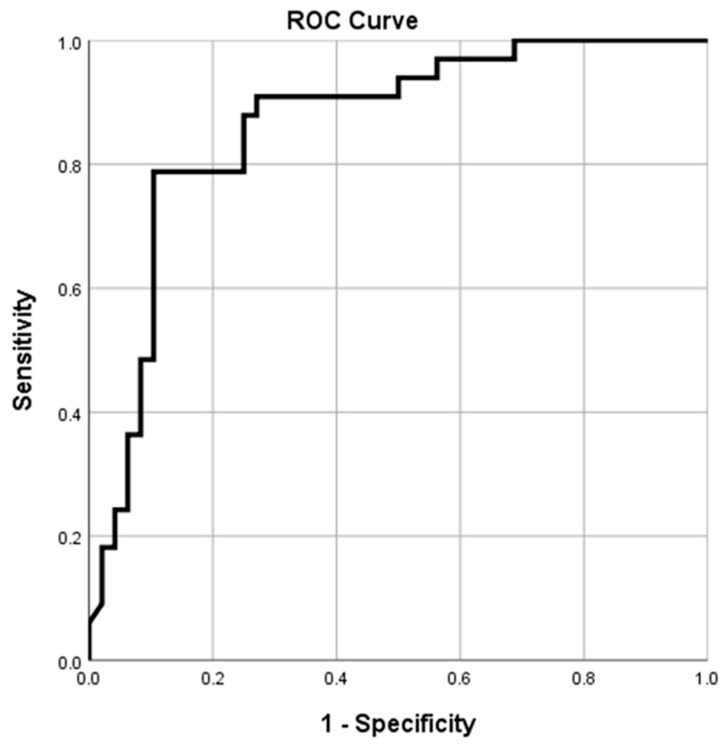
The receiver operating characteristic (ROC) plot of handgrip strength (HGS) and peak inspiratory flow rate (PIFR) for Turbuhaler. The best cutoff value of HGS for Turbuhaler is 31.9 kg, with the area under the ROC curve of 0.862 (95% CI; 0.779–0.945, *p* < 0.001), 79% sensitivity, and 90% specificity.

The area under the ROC curve of 0.892 (95% CI; 0.824–0.961, *p* < 0.001) for the best cutoff value of HGS for Accuhaler was 26.8 kg, with 82% sensitivity and 84% specificity (Table 4, Figure 4). The best cutoff value of HGS for Turbuhaler was 31.9 kg, with the area under the ROC curve of 0.862 (95% CI; 0.779–0.945, *p* < 0.001), 79% sensitivity, and 90% specificity (Table 5, Figure 5).

## 4. Discussion

Based on the results of our study, there is a significant positive correlation between HGS and PIFR for Accuhaler and Turbuhaler DPIs, suggesting HGS may be a valuable test in the assessment of the ability of the respiratory system to create adequate PIFR. To our knowledge, this is the first study to identify a HGS cutoff value for predicting the adequacy of PIFR in patients with clinically stable COPD.

A previous study of 44 clinically stable patients with COPD by Tsuburai et al. [21] showed a significant positive correlation between HGS and PIFR for Accuhaler (r = 0.326, *p* = 0.031) and Turbuhaler (r = 0.328, *p* = 0.030). This study differed from our study in that the investigators did not derive a prediction equation for PIFR considering both genders and all stages of disease, and they did not identify a HGS cutoff value associated with suboptimal PIFR. Our study showed higher correlation coefficients of HGS and PIFR (r = 0.591 for Accuhaler and r = 0.614 for Turbuhaler) than the study by Tsuburai [21], probably because of the larger number of patients in our study and the broader range of HGS. A study of 75 hospitalized patients with acute exacerbation of COPD by Samarghandi et al. [3] demonstrated that HGS was significantly correlated with Accuhaler PIFR. Patients in the suboptimal PIFR group had significantly lower HGS than those in the optimal PIFR group, and each kilogram increase in HGS was associated with a 0.5 L/min increase in PIFR [3]. Similarly, our study demonstrated that the suboptimal PIFR group had significantly lower HGS than the optimal group in both Accuhaler and Turbuhaler. These results suggest that HGS might be able to predict the efficiency of inhaled drug delivery in COPD patients with or without exacerbation.

There were variations of the prevalence of suboptimal PIFR in patients with COPD in previous studies. The study by Sharma et al. revealed that the proportion of suboptimal PIFR for Accuhaler DPI was 32% at discharge following hospitalization for exacerbation of COPD, but this group had no difference in incidence of all-cause rehospitalization up to 180 days compared to the optimal PIFR group [9]. A study by Ghosh et al. showed that 40% of outpatients with COPD were unfit to use prescribed inhalers. Suboptimal PIFR (PIFR < 60 L/min) was 44% of low-medium resistance DPIs (mimicking Accuhaler and Ellipta), and PIFR < 30 L/min was 32% of high resistance DPI (mimicking Handihaler) [10]. A study by Harb et al. [11] showed that the prevalence of suboptimal PIFR of any resistance representative of a specific inhaler was 44.44% in COPD patients before hospital discharge. In our study, the prevalence of suboptimal PIFR was 38.3% in Accuhaler and 59.3% in Turbuhaler. All of these studies suggest that many COPD patients might not generate adequate inspiratory force to overcome prescribed DPI resistance. These results suggest that Accuhaler might be more suitable for certain COPD patients than Turbuhaler, because the prevalence of suboptimal PIFR for Accuhaler was lower than that for Tubuhaler. Interestingly, a previous study also demonstrated that the mean peak inspiratory flow for Accuhaler was significantly higher than it was for Turbuhaler [22]. 

Several previous studies demonstrated that one factor associated with suboptimal PIFR was female sex [9,10,11]. A literature review by Ghosh et al. [8] found that female sex and older age were factors associated with lower PIFR. In concordance with these findings, our results showed that suboptimal PIFR was more common in female than in male patients, and the mean PIFR was significantly lower in female patients than it was in men. Furthermore, we found that other factors associated with suboptimal PIFR were older age, higher breathing frequency, more inhaled SABD use, higher CAT and mMRC scores, lower HGS, shorter height, and lower pulmonary function. These indicate that a higher symptom burden affects patients’ health status and their inspiratory force, leading to suboptimal PIFR.

Our study revealed that the best HGS cutoff values for predicting optimal PIFR for Accuhaler and Turbuhaler had a large area under the ROC curve with high sensitivity and specificity. Therefore, HGS might be applied as a predictive tool for optimal PIFR in clinically stable COPD patients being considered for prescription of Accuhaler and Turbuhaler devices. Moreover, these cutoff values might be particularly useful in elderly patients with COPD because the mean age of participants in our study was 73 years. Older patients with stable COPD were commonly found in several observational studies, including a study in the same research center by Saiphoklang N et al. [23]. A previous study by Fronhofen et al. [24] demonstrated that a threshold HGS value of 10 kg could predict inspiratory flow achievement for Turbuhaler in hospitalized elderly patients with COPD. In contrast to that previous study, our study found that the cutoff value of HGS was higher (31.9 kg) for predicting the optimal Turbuhaler PIFR. The reason may be because the participants in the study by Fronhofen et al. [24] were even older than those in our study (mean age of 81 versus 73 years). Although patient settings may differ, our study suggests that HGS can be applied to all elderly patients with COPD by using the predicting equations. However, assessment of HGS may be limited by the cooperation of patients and by hand abnormalities.

Furthermore, HGS could also predict clinical outcomes in mechanically ventilated patients, including COPD patients (12% of the study subjects) [25]. A previous study by Strandkvist VJ et al. revealed that COPD patients with heart disease had lower HGS than those without heart disease [26]. These patients with GOLD spirometric stages 3–4 (severe to very severe airflow limitation) had lower HGS than those without COPD [26]. 

Clinical applications are proposed from our study. HGS might be a reasonable alternative to PIFR to evaluate the adequacy of inspiratory force for DPIs. The best cutoff values of HGS for optimal Accuhaler and Turbuhaler PIFR are 26.8 kg and 31.9 kg, respectively.

This study has certain limitations. Firstly, a small sample size of the population was used in this study. Therefore, some explanatory variables for calculated PIFR using regression coefficients might not be as precise as indicated by the wide 95% CI, and some variables might be outliers in the outcome variables. Moreover, only 8% of participants were COPD group D; thus, it might not have a realistic association between HGS and PIFR in clinical practice. Secondly, the study was conducted in a single research center in Thailand; the results might not be applicable to other ethnicities or countries. Thirdly, the participants were clinically stable COPD patients without acute exacerbation, and none of them used home oxygen therapy or home invasive or non-invasive mechanical ventilation. Therefore, the cutoff value of HGS might not be applicable for more severe and debilitated COPD patients, such as those with exacerbations, home oxygen therapy or mechanical ventilation, and other obstructive lung diseases. Fourthly, there were 14% women in this study, which may be a substantial under-representation of women with COPD that could impact generalizability. We did not collect data on patients with sarcopenia or data on muscle mass and function, e.g., walking speed. Therefore, we could not speculate that gender differences independently influence PIFR, especially in female Turbuhaler DPI users. Lastly, these cutoff values were analyzed to apply only to patients who potentially use Accuhaler and Turbuhaler. Consequently, they may not apply to patients who use other types of inhaler devices. A future study is required to determine the correlation between HGS and optimal PIFR for other types of inhaler devices and in different settings of patients, such as stable COPD patients with home oxygen therapy or mechanical ventilation, or patients with other pulmonary diseases.

## 5. Conclusions

HGS was positively correlated with Accuhaler and Turbuhaler PIFR in clinically stable COPD patients, especially in the group with pronounced symptoms without frequent exacerbations. The prevalence of suboptimal PIFR, defined by PIFR < 60 L/min, was found in up to half of the patients. The suboptimal group had more females, older patients, higher breathing frequency, more inhaled SABD users and doses, higher CAT and mMRC scores, lower HGS, shorter height, and worse pulmonary function compared to the optimal groups. HGS may be a predictive tool for determining the efficacy of inhaler drug delivery.

## Figures and Tables

**Table 1:** Baseline characteristics of clinically stable patients with chronic obstructive pulmonary disease.

Characteristic	N = 81
Age, years	73.3 ± 8.9
Male	70 (86.4)
Body mass index, kg/m^2^	22.5 ± 3.9
Active smoking	4 (4.9)
Smoking, pack-years	24.9 ± 22.2
Dominant right hand	72 (88.9)
Maximal Accuhaler PIFR, L/min	63.7 ± 18.9
Maximal Turbuhaler PIFR, L/min	52.4 ± 15.4
HGS, kg	29.2 ± 8.0
Comorbidity	
Hypertension	44 (54.3)
Diabetes mellitus	21 (25.9)
Coronary artery disease	14 (17.3)
Congestive heart failure	5 (6.2)
Atrial fibrillation	4 (4.9)
Lung cancer	4 (4.9)
Bronchiectasis	4 (4.9)
Depression	1 (1.2)
Vital sign	
Systolic blood pressure, mmHg	128.4 ± 21.5
Diastolic blood pressure, mmHg	71.3 ± 12.2
Pulse rate, beats/min	81.4 ± 14.3
Breathing frequency, breaths/min	17.0 ± 1.9
Oxygen saturation, %	96.4 ± 4.0
Spirometric data	
Post-BD FEV_1_, L	1.45 ± 0.60
Post-BD FEV_1_, % predicted	65.3 ± 23.7
Post-BD FVC, L	2.50 ± 0.90
Post-BD FVC, % predicted	82.7 ± 22.1
Post-BD FEV_1_/FVC, %	58.0 ± 11.0
Functional performance	
CAT scores	9.5 ± 6.5
CAT score < 10	40 (49.4)
CAT score ≥ 10	41 (50.6)
mMRC scores	1.5 ± 1.1
mMRC < 2	42 (51.9)
mMRC ≥ 2	39 (48.1)
Spirometry grading	
1	32 (39.5)
2	24 (29.6)
3	16 (19.8)
4	9 (11.1)
GOLD classification	
A	35 (43.2)
B	39 (48.1)
C	0 (0)
D	7 (8.6)
Medication	
SABD	25 (30.9)
SABD dose, puffs/day	1.0 ± 1.9
Inhaled LABA	2 (2.5)
LAMA	43 (53.1)
LABA/LAMA	21 (25.9)
ICS/LABA	36 (44.4)
Xanthine	30 (37.0)
Phosphodieterase-4 inhibitor	4 (4.9)
Oral beta-2 agonist	5 (6.2)
Azithromycin	5 (6.2)
Acetylcysteine	36 (44.4)

Data shown n (%) or mean ± SD BD = bronchodilator, CAT = COPD assessment test, FEV_1_ = force expiratory volume in 1 s, FVC = forced vital capacity, GOLD = Global Initiative for Obstructive Lung Disease, HGS = hand grip strength, ICS = inhaled corticosteroid, LABA = long-acting beta2 agonist, LAMA = long-acting muscarinic antagonist, mMRC = modified Medical Research Council, PIFR = peak inspiratory flow rate, SABD = short-acting bronchodilator.

**Table 2:** Comparison of characteristics of clinically stable COPD patients between the optimal and the suboptimal PIFR groups for Accuhaler and Turbuhaler.

Variable	Accuhaler			Turbuhaler		
	Optimal	Suboptimal	*p*-Value	Optimal	Suboptimal	*p*-Value
Patients	50 (61.7)	31 (38.3)	NA	33 (40.7)	48 (59.3)	NA
Maximal PIFR, L/min	75.6 ± 12.3	44.7 ± 9.9	<0.001	67.4 ± 6.7	42.2 ± 10.5	<0.001
HGS, kg	33.2 ± 6.9	22.8 ± 4.7	<0.001	35.1 ± 6.3	25.3 ± 6.4	<0.001
Gender						
Male	49 (98.0)	21 (67.7)	<0.001	33 (100)	37 (77.1)	0.002
Female	1 (2.0)	10 (32.3)		0 (0)	11 (22.9)	
Age, years	70.0 ± 8.8	78.7 ± 6.2	<0.001	67.9 ± 8.6	77.0 ± 7.2	<0.001
Height, cm	165.7 ± 6.1	158.6 ± 8.0	<0.001	166.8 ± 5.1	160.4 ± 8.1	<0.001
BMI, kg/m^2^	22.8 ± 4.0	22.0 ± 3.7	0.372	23.2 ± 3.6	22.1 ± 4.0	0.190
Active smoking	3 (6.0)	1 (3.2)	1.000	2 (6.1)	2 (4.2)	1.000
Smoking, pack-years	27.5 ± 22.4	20.7 ± 21.7	0.182	25.3 ± 18.1	24.6 ± 24.9	0.892
Dominant hand						
Left	7 (14.0)	2 (6.5)	0.471	3 (9.1)	6 (12.5)	0.731
Right	43 (86.0)	29 (93.5)		30 (90.9)	42 (87.5)	
Comorbidity						
Diabetes mellitus	12 (24.0)	9 (29.0)	0.615	8 (24.2)	13 (27.1)	0.774
Hypertension	28 (56.0)	16 (51.6)	0.700	21 (63.6)	23 (47.9)	0.163
Coronary artery disease	6 (12.0)	8 (25.8)	0.110	5 (15.2)	9 (18.8)	0.674
Congestive heart failure	3 (6.0)	2 (6.5)	1.000	2 (6.1)	3 (6.3)	1.000
Atrial fibrillation	2 (4.0)	2 (6.5)	0.635	2 (6.1)	2 (4.2)	1.000
Lung cancer	1 (2.0)	3 (9.7)	0.154	1 (3.0)	3 (6.3)	0.642
Bronchiectasis	1 (2.0)	3 (9.7)	0.154	0 (0.0)	4 (8.3)	0.142
Vital sign						
SBP, mmHg	126.9 ± 24.4	130.9 ± 15.9	0.424	130.2 ± 16.0	127.3 ± 24.7	0.554
DBP, mmHg	73.0 ± 12.9	68.8 ± 10.7	0.126	74.6 ± 13.0	69.1 ± 11.2	0.044
Pulse rate, beats/min	81.3 ± 14.2	81.6 ± 14.6	0.932	80.7 ± 13.2	81.9 ± 15.1	0.713
Respiratory rate, breaths/min	16.6±1.8	17.6±1.96	0.031	16.3±1.6	17.4±2.0	0.007
SpO_2_, %	96.6±4.5	96.1±3.1	0.578	96.3±5.3	96.5±2.8	0.837
Spirometry data						
Post-BD FEV_1_, %	70.6 ± 20.2	56.8 ± 26.6	0.010	75.2 ± 18.4	58.5 ± 24.7	0.001
Post-BD FVC, %	88.5 ± 18.1	73.4 ± 24.9	0.002	93.0 ± 17.9	75.7 ± 22.0	<0.001
Spirometric grade 3 and 4	12 (24.0)	13 (16.0)	0.089	5 (15.2)	20 (41.7)	0.011
Functional performance						
CAT scores	7.8 ± 6.3	12.2 ± 6.0	0.003	5.5 ± 5.1	12.2 ± 5.9	<0.001
CAT ≥ 10	20 (40.0)	21 (67.7)	0.015	7 (21.2)	34 (70.8)	<0.001
mMRC scores	1.2 ± 1.1	2.0 ± 0.9	0.001	0.9 ± 0.9	2.0 ± 1.0	<0.001
mMRC ≥ 2	17 (34.0)	22 (71.0)	0.001	6 (18.2)	33 (68.8)	<0.001
GOLD group D	5 (10.0)	2 (6.5)	0.702	1 (3.0)	6 (12.5)	0.136
Medication						
SABD	11 (22.0)	14 (45.2)	0.028	5 (15.2)	20 (41.7)	0.014
SABD dose, puffs/day	0.6±1.3	1.6±2.6	0.043	0.4±1.1	1.4±2.3	0.008
LABA	1 (2.0)	1 (3.2)	1.000	1 (3.0)	1 (2.1)	1.000
LAMA	28 (56.0)	15 (48.4)	0.505	15 (45.5)	28 (58.3)	0.254
LABA/LAMA	10 (20.0)	11 (35.5)	0.122	10 (30.3)	11 (22.9)	0.456
ICS/LABA	25 (50.0)	11 (35.5)	0.201	14 (42.4)	22 (45.8)	0.762
Theophylline	10 (20.0)	5 (16.1)	0.663	6 (18.2)	9 (18.8)	0.948
Doxophylline	7 (14.0)	8 (25.8)	0.184	7 (21.2)	8 (16.7)	0.605
Roflumilast	3 (6.0)	1 (3.2)	1.000	1 (3.0)	3 (6.3)	0.642
Oral beta2 agonist	3 (6.0)	2 (6.5)	1.000	2 (6.1)	3 (6.3)	1.000
Azithromycin	2 (4.0)	3 (9.7)	0.366	0 (0)	5 (10.4)	0.076
Acetylcysteine	18 (36.0)	18 (58.1)	0.052	11 (33.3)	25 (52.1)	0.095

Data shown n (%) or mean ± SD BD = bronchodilator, BMI = body mass index, CAT = COPD assessment test, DBP = diastolic blood pressure, FEV_1_ = force expiratory volume in 1 s, FVC = forced vital capacity, GOLD = Global Initiative for Obstructive Lung Disease, HGS = hand grip strength, ICS = inhaled corticosteroid, LABA = long-acting beta2 agonist, LAMA = long-acting muscarinic antagonist, mMRC = modified Medical Research Council, PIFR = peak inspiratory flow rate, SABD = short-acting bronchodilator, SBP = systolic blood pressure, SpO_2_ = oxygen saturation.

**Table 3:** Correlation between handgrip strength with peak inspiratory flow rate for Accuhaler and Turbuhaler.

Factor	Correlation Coefficient	*p*-Value
Accuhaler PIFR	0.591	<0.001
Turbuhaler PIFR	0.614	<0.001

PIFR = peak inspiratory flow rate.

**Table 4:** Linear regression analysis for peak inspiratory flow rate and hand grip strength adjusted by age, gender, height, forced vital capacity, and short-acting bronchodilator dose.

Variables	Regression Coefficients	95% CI of Coefficients	*p*-Value
Accuhaler			
Intercept	−30.340	−114.534, 53.853	0.475
Hand grip strength, kg	0.274	0.132, 1.170	0.015
Age, years	−0.206	−0.847, −0.025	0.038
Height, cm	0.219	0.063, 1.019	0.027
Forced vital capacity, %	1.019	0.099, 0.389	0.001
Short-acting bronchodilator dose, puffs/day	−0.148	−3.118, 0.238	0.091
Turbuhaler			
Intercept	56.196	20.715, 91.676	0.002
Hand grip strength, kg	0.321	0.210, 1.032	0.004
Female	−0.196	−16.718, −0.850	0.030
Age, years	−0.224	−0.717, −0.058	0.022
Forced vital capacity, %	0.304	0.096, 0.330	0.001
Short-acting bronchodilator dose, puffs/day	−0.147	−2.508, 0.173	0.087

**Table 5:** Cutoff values of hand grip strength for predicting optimal Accuhaler and Turbuhaler PIFR.

Variable	Cutoff Value	AUC	95% CI	Sensitivity (%)	Specificity (%)	PPV (%)	NPV (%)	*p*-Value
HGS for Accuhaler, kg	26.75	0.892	0.824–0.961	82.00	83.90	89.14	74.32	<0.001
HGS for Turbuhaler, kg	31.90	0.862	0.779–0.945	78.80	89.60	83.87	86.03	<0.001

AUC = area under the ROC curve, CI = confidence interval, HGS = hand grip strength, NPV = negative predictive values, PIFR = peak inspiratory flow rate, PPV = positive predictive values.

## Data Availability

The data supporting the results of this study are available within the article.
